# Control of Hæmorrhage by Ipecac

**Published:** 1871

**Authors:** William Martin

**Affiliations:** Billingsville, Indiana


I.—*Control of Hæmorrhage by Ipecac.* By WILLIAM MARTIN, M.D., M. R. C. S., Billingsville, Indiana.

---

*April* 1, 1871.—At 9 A. M., was called to see my friend, Mr. W. D., a farmer, residing in the village where I practice, and upon inquiry found that, entering his barn, with a long, roughened bamboo-stemmed pipe in his mouth, some hogs ran against his legs and he was forcibly thrown aginst a post, the pipe-stem entering his right tonsil to the apparent depth of two-thirds of an inch. Having examined the injury to tonsil, and remembering the close apposition of the internal carotid artery, I resolved, as there was then little hæmorrhage, on doing nothing but apply perchloride of iron on a sponge, being afraid if I made much exploration that I should cause bleeding from the anastomosing arteries, and thus cause trouble. No bad symptoms occurred that day, my patient only complaining of soreness in the muscles of the neck and face, caused, no doubt, by violence to ramifying branches of the fifth nerve.

*April* 2*d.*—I was sent for in a hurry at 6 A. M., and found my patient in a state of prostration and fear. Pulse 104, neck and tongue swollen, and positive jutting arterial hæmorrhage from back and upper surface of wound, which, altogether, presented a most discouraging and alarming appearance. I should here state that headache was much complained of in the region of lateral meningeal arteries. Ordered twenty grains of hydrate chloral, and gargle containing one part in four of tinct. ferri muriatis.

3 P. M.—No change, except complained of suffocation, and on examining wound (and being *quite certain* that hæmorrhage was not from carotid) I gave two grain doses of ipecac, every hour, till third dose caused *gentle vomiting*, and with it immediate contraction of tonsil (thus lessening wound,) and causing hæmorrhage at once to stop.

10 P. M.—Anxious to let well alone, and kind Nature do her part, I left him comfortable as was possible under pains and losses, only administering twenty-five drops Battley's solution (liq. opii. sed.;) well cautioning his wife to send for me if any change should occur, especially bleeding from mouth.

*April 3d,* 8 A. M.—Found my patient in same state of fever, much headache, and complaining of acute pains in region of sterno-mastoid, omo-hyoid, and scaleni muscles. Ordered full dose of ol. ricini, which, having acted, reduced *all* pains and partly the suffocating sensation, but left him (a powerful and naturally healthy man) greatly reduced and depressed. Ordered table-spoonful of whisky every hour, and administered as medicine:

℞.—Ammon. carb., ℈ij.

Tinct. cinchon. co., ℥ ss.

Aqua. ad., ℥ viij.

Ft. mistura et capiat cochleare ij 4tis horis.

9 P. M.—Found him, as his own familiar tongue expressed it, "bully," and, continuing the astringent gargle and tonic for a few days, my patient was left in his wife's hands to nourish, he having, indeed, had a narrow escape, and I spared a good patient who is now in perfect health, and I believe smokes the same pipe.

*Remarks.*—I report this case in honor of chloral and ipecac, and I am quite convinced, from the experience of nineteen years, that the theory and practice of my lamented teacher, Sir William Lawrence, Bart., that in such cases, where capillary hæmorrhage occurs, and the position of main artery dangerous, that after the administration of *small doses* of ipecac, until a *gentle* vomit is caused, natural plugging follows contraction of tissues, and safety is insured, for in such cases and under such treatment, muscular contraction *never* fails. I have tried it many times (even so late as last week) in obstinate contraction of uterus, and never experienced ill effects.—*New York Medical Journal.*

